# Why doesn’t integrated care work? Using Strong Structuration Theory to explain the limitations of an English case

**DOI:** 10.1111/1467-9566.13398

**Published:** 2021-11-06

**Authors:** Gemma Hughes, Sara E. Shaw, Trisha Greenhalgh

**Affiliations:** ^1^ Nuffield Department of Primary Care Health Sciences University of Oxford Oxford UK

**Keywords:** ageing, case study, chronic conditions, ethnography, hospital admissions avoidance, integrated care, Strong Structuration Theory

## Abstract

Integrated care is an aim and a method for organising health and care services, particularly for older people and those with chronic conditions. Policy expects that integrated care programmes will provide person‐centred coordinated care which will improve patient or client experience, enable population health, prevent hospital admissions and thereby reduce costs. However, empirical evaluations of integrated care interventions have shown disappointing results. We analysed an in‐depth case study using Strong Structuration Theory to ask: how and why have efforts to integrate health and social care failed to produce desired outcomes? In our case, integrated case management and the creation of cost‐saving plans were dominant practices. People working in health and social care recursively produced a structure of integrated care: a recognised set of resources created by collective activities. Integrated care, intended to help patients manage their long‐term conditions and avoid hospital admission, was only a small part of the complex network that sustained patients at home. The structures of integrated care were unable to compensate for changes in patients’ health. The result was that patients’ experiences remained largely unaffected and hospital admissions were not easily avoided.

## INTRODUCTION

Integrated care is both a common aim for, and a process adopted by, health systems around the world. It can be defined as an organising principle for health and care delivery and a set of initiatives and service models aimed at realising person‐centred coordinated care in the face of population challenges such as ageing and increasing multi‐morbidity (Damarell et al., 2020). Endorsed by the World Health Organization as a framework for the redesign of health systems, integrated care is considered to be essential in improving care for people with chronic conditions who require ongoing care and support (World Health Organization, 2016). As such, integrated care is expected by policy‐makers and healthcare improvement institutions to contribute to achieving the ‘triple aim’ of healthcare—better outcomes, experiences and use of resources.

Health services research on integrated care has focussed extensively on evaluation. In the UK, findings have been disappointing. Patients have not consistently reported benefits, and emergency hospital admissions have risen rather than, as planned, fallen. An important feature of the literature on integrated care is the broad range of activities and concepts that are associated with the term. The imprecision of ‘integrated care’ explains, in part, the lack of demonstrable outcomes due to methodological challenges of comparing diverse models and of evaluating complex interventions. Further, patients are not always aware that it is integrated care that they are receiving, even when they report satisfaction with their experiences (Gowing et al., 2016; Greenfield et al., 2014). Studies of integrated care as a social phenomenon, rather than as an intervention to be evaluated, take a more theoretical and critical approach to show how integrated care is experienced by patients as a complex trajectory (Allen, Griffiths and Lyne, 2004), its consequences as a form of governmentality (Pickard, 2009) and the somewhat slippery nature of the policy of integrated care as it manifests in programmes, workstreams and governance arrangements (Jones, 2018). Integrated care is understood as produced from the dynamics between people, structures, systems and ideas (Williams and Sullivan, 2009, Embuldeniya et al., 2018) and from the intersection of governance frameworks (Jones, 2017). Integrated care has also been shown to contribute towards the social processes of healthcare (Tousijn, 2012, Lusardi and Tomelleri, 2018). The premise that integrated care is a necessary goal and process for health system improvement remains unchallenged by these bodies of evidence. Instead, explanatory models account for and predict health system success as both a process and outcome of integrated care, generating a normative view of its inherent benefits (Hughes, Shaw and Greenhalgh, 2020).

The case for taking a sociological approach to service changes such as integrated care has been well made (Fraser et al., 2019). We contribute to this body of literature by connecting the normative body of health services research with the sociological literature by theorising how integrated care often fails to improve patients’ experiences and service outcomes. We ask: how have efforts to integrate health and social care failed to affect the desired outcomes of improved patient experiences and reduced hospital admissions? We use Strong Structuration Theory (SST) to analyse an empirical case to find that the work of integrated care comprises a set of practices that produce a meso‐level structure. Here, we conceive of *structure* as a recognisable set of resources (including ideas, roles and texts) that are created from collective (involving multiple people) activities and intentions, and which shape further collective activities. As a *meso‐*level structure, integrated care is produced as a concept with a degree of abstraction but situated in a particular set of circumstances. Guided by SST, we understand the relationship between integrated care and collective working practices as being recursive, with integrated care being both a condition for, and an outcome of, action. In other words, as people work to integrate care they create the conditions within which care can be integrated (or not). We argue that the structure of integrated care forms a ‘lens’ that shapes the actions of professionals but which forms only a small part of the complex network of resources that patients mobilise to sustain their lives at home.

The remainder of this paper is organised as follows: first, we introduce Strong Structuration Theory (SST) and describe the research setting and methods of an empirical case of integrated care. Then, we set out findings of how our case (a partnership of health and care organisations working to integrate care) emerged from an interplay between policy and organisational conditions. We analyse how a meso‐level structure of integrated care was recursively produced by health and social care professionals and patients’ experiences of that structure. We conclude that, as a set of resources, integrated care is not a structure that consistently affects patients’ experiences and outcomes.

## APPLYING STRONG STRUCTURATION THEORY (SST) TO INTEGRATED CARE

Structuration theory is concerned with the relationships between structures (social realities that exist independently of individual actors) and agency (how individuals exert their own choices and actions). Giddens (1984) considers these relationships to be recursive: individuals’ subjective reality (including their actions, values and judgement) produce and reproduce macro structures, whilst being constrained and shaped by those structures. At the heart of structuration theory is the idea of the duality of structures, which are understood to be both mediators of practices (enabling, facilitating or constraining and shaping what people say and do) and outcomes of practices (structures are produced and reproduced by these practices). Stones extends Giddens’s structuration into Strong Structuration Theory (SST) by shifting the analytic focus from abstract processes of structuration (what Stones called an ‘ontology in general’), to ‘ontology in situ’. Stones conceives of four components of the recursive relationship between structure and agency that can be studied empirically: external social structures (conditions for action), internal social structures (what agents know—or believe they know—about the social world), active agency (what agents do in particular social situations) and outcomes (both short term, affecting the immediate situation, and long term, feeding into continuity or change in social structures). These four components make up the ‘quadripartite’ analytic lens of SST (Stones, 2005). In the healthcare field, SST has been used to study the phenomenological experiences of assisted living technology (Greenhalgh et al. 2013), healthcare governance (Bodolica et al. 2016), digital interactions in general practice (Assing et al., 2021) and—with an added technology component—the implementation of information technology systems in health care (Greenhalgh and Stones 2010).

We found SST to be particularly relevant to analysing the specificity and scale of the practices found in our empirical case as we traced the service changes of integrated care across a ‘field of practice’ (Fraser et al., 2019); that is, we considered how the conditions for, and practices of, integrated care for individual patients were shaped by changes in organisational arrangements as well as evolving national policies. We used SST as a framework to examine the conditions that produced structures of integrated care, how these structures were recursively produced by agents (commissioners and healthcare professionals) and how the outcomes of avoiding hospital admissions were shaped (or not) by the dynamics between the structures of integrated care and the actions of patients and professionals. We therefore extend existing analysis of structure and agency in integrated care in three ways by: including patients’ actions and experiences in our empirical investigation and theoretical analysis, considering how meso‐structures of integrated care are produced and, situating our analysis of integrated care in a field of practices which connects patients to policy directives.

## RESEARCH SETTING AND METHODS

In the UK, integrated care has been a consistent element of health policy for decades, resulting in a series of pilots, programmes and other temporary projects aimed at improving health outcomes for different groups of patients (including people with diabetes, older people and those at high risk of hospital admission), reducing unnecessary hospital admissions and containing healthcare costs (Windle et al., 2009; Roland et al., 2012;, Harris et al., 2013, NHS England, 2016, Erens et al., 2017). More recently, the scope of integrated care in England has extended from organisational changes to those required to integrate *systems:* groups of organisations in defined locations (NHS England, 2019, Department of Health and Social Care, 2021). We therefore conceptualise integrated care in the UK as being performed at and influencing activities at different scales: the macro actors and actions of national policy (decisions which affect many people such as allocation of funding, setting of targets or promoting particular models of care); meso networks of organisations creating common strategies and patterns of working; and the micro practices of individual professionals and patients. Considering integrated care as a distributed field of practices, with actors connected through policies, organisational strategies and targeted programmes allowed us to analyse how organisational and policy processes produced integrated care, and how professionals and patients contextualised and interpreted integrated care.

We took for our case a group of NHS organisations and councils (pseudonymised as ‘the Partnership’) and their collaborative work on an integrated care strategy across a defined geographical area (Box 1). The scope of this field of practices stretched from the micro practices of case management to the meso‐macro structures of NHS strategic and financial planning where organisational relationships were guided by, and informed, national policy and resourcing decisions. We used ethnographic methods to access the empirical ground of the dynamics between individual patients and professionals and organisational strategy and decision‐making. Participant observation provided access to strategy development and financial planning meetings, case management discussions and patients’ experiences at home and in healthcare settings.

BOX 1 **The Partnership**

The Partnership comprised the NHS organisations (acute hospitals, community health services, primary care providers and clinical commissioning groups) and councils that covered a population of around 700,000 in an area that stretched from the edges of an inner‐city to the suburbs. The fluidity of NHS organisational arrangements became apparent when the Partnership proved to be a temporary configuration: Sustainability and Transformation Plans/Partnerships were established across larger geographical areas in 2016, which have since been superseded by Integrated Care Systems.

There were significant differences between the involvement of councils and NHS organisations in the Partnership. NHS organisations were collectively required to produce unified plans for the funding and provision of health services for the combined population. Councils did not share NHS administrative boundaries. As discrete statutory bodies, councils remained separately accountable to their democratically elected members and were represented by senior executives whose responsibilities included Adult Social Care. Councils in the Partnership had little in common with each other politically, finding common purpose in their concerns to influence NHS services to see improvements in the quality of care for their constituents. With no statutory powers, the Partnership itself was a strategic alliance of executives from the health and social care organisations in the area who agreed to work together to address common areas of concern, such as delayed hospital discharges.

Prior to the creation of the Partnership, a number of strategies and projects reflecting a history of national and local integrated care initiatives and programmes had been introduced in the area, including joint appointments between health and social care, integrated community teams, case management and pooled budgets.

Data (extensive fieldnotes, interviews and documents) were generated during 3 years of fieldwork which included participant’s observation by one of us (GH) of 20 patients’ experiences of receiving integrated care in their homes and healthcare settings (length of contact varied between individual patients from 4 to 19 months) and of the practices of commissioning and planning integrated health and care (access offered by GH’s concurrent role as a CCG commissioner in the case throughout fieldwork).

We categorised empirical data into: patient experience, professional practice, commissioning, and policy, planning and legislative context (Table 1). We received ethical approval from NRES Committee London: Camden & Islington Research Ethics Committee (Ref 13/LO/1610). We have anonymised our case by applying pseudonyms to individuals and organisations.

**TABLE 1:** summary of data and analysis

Category of data	Dataset	Synthesis (1)	Synthesis (2)	Analysis
Patient experience	20 research participants (93 semi‐structured interviews/visits and observations with patients and carers in home and healthcare settings)	20 individual patient case summaries	Trans‐contextual analysis of case management	Analysis of processes of structuration: 1. Identifying conditions for action, internal and external structures, actions and outcomes. 2. Analysing relationships between the above
Professional practice	13 interviews with 10 professionals (community nurses, social workers, liaison officers), 13 observations including shadowing and observations of inter‐professional team meetings	chronology of organisational initiatives to integrate care		
Commissioning	8 interviews with 8 participants (senior NHS and council officials, commissioners and managers), fieldnotes from observations over 3 years, review of 191 documents (consultation documents, finance and activity data, contractual agreements)			
Policy, planning and legislative context	2 interviews with policy‐makers and analysis of 56 contemporary and historical policy, guidance and legislative document	narrative and discourse analysis of policy		

Analysis comprised a series of linked phases. First, we constructed different accounts of integrated care including: patient case summaries; a chronology of organisational initiatives to integrate care; and narrative and discourse analysis of policy. We synthesised these accounts in a transcontextual analysis of case management (Rampton, Maybin and Roberts, 2015), creating a composite narrative of the practices associated with integrated case management (ICM), following Jarzabkowski et al.’s (2014) method of merging multiple observations into a single, typical narrative. The composite narrative of ICM (Box 2) allowed us to examine how events encountered during fieldwork connected with broader social structures, institutions and ideologies across the field of practices of integrated care. We distinguish between those policies, events and histories that preceded the development of a specific integrated care strategy by the Partnership and a corresponding set of actions that created a local structure of integrated care. We define the policies and events which preceded the Partnership’s strategy as external conditions for action.

Central to the composite narrative were a series of events where we observed the dynamics of structures, agency and outcomes in situ. We selected three such sites (development of finance plans, ICM meetings and patient’s daily lives) to undertake a quadripartite analysis of the duality of the structure of integrated care by examining dynamics of the external structures (conditions of action autonomous of individual actors), internal structures (specific orientation and more general understanding), active agency (how people chose to respond) and outcomes (internal and external structures and events) of each of these events as integrated care structures were produced.

## FINDINGS

We found that external conditions for action emanated from the macro context of national policies and discourses to interplay with the meso context of the case and so create the conditions for the local practice and experience of integrated care. We found that the meso‐level structure of integrated care created in the Partnership was part of the conditions for action for the practices of community‐based health and care professionals, that is, knowledge of the strategy and resources made available by the strategy, shaped how professionals acted. We identified two distinct sets of practices by professionals that recursively produced the structure of integrated care: financial planning processes and the enactment of a case management model of care. Further, the outcomes of the professionals’ practice contributed towards (but failed to determine) the conditions of patients managing multiple long‐term conditions at home.

## External conditions for integrated care

External conditions that made a Partnership strategy for integrated care possible and necessary were produced from an interplay between national policies and situated organisational histories. National trends of reducing hospital admissions, numbers of hospital beds and restricting expenditure on health and social care manifested in detailed plans in our specific case. Legislative changes affecting NHS organisations, combined with local patterns of services, also influenced how the Partnership was formed, laying the ground for an integrated care strategy. The formation of the Partnership was a situated example of collective strategic leadership (Denis et al., 2001), whilst the creation of the strategy represented a ‘common logic’ (Tuohy, 1999) found in similar strategies developed elsewhere in England.

The trend in UK health policy to reduce reliance on institutional and hospital care and move ‘care closer to home’ (NHS England, 2014, Department of Health, 2006) is interwoven with policies aimed at avoiding inappropriate and inefficient hospital admissions for older people (Audit Commission, 1997). The preferred model is proactive preventative care in the community, codified in case management which had the specific aim of preventing hospital admissions (Department of Health, 2005). Specific concerns about the acute hospitals in our case (regulators had reported failures of care and financial management) led to a convergence of national and local concerns. The senior leaders of NHS organisations (providers and commissioners) concluded in 2010 that hospital services should be reconfigured, reducing hospital beds and closing an A&E. Despite being aligned with broader national trends of reducing hospital beds, reconfiguration plans were contentious among local councillors and public and patient representatives who agreed that improvements were needed but interpreted the strategy to reduce hospital beds as a reduction in health services. The Partnership sought to counter these concerns with proposals to strengthen community‐based health care.

A national context of constrained expenditure on health and social care posed particular problems in our case. Pessimistic financial forecasts led to Partnership saving plans aimed at balancing growing demand for services against diminishing funding allocations. Presented in policy and grey literature as being able to relieve pressure on hospital beds, integrated care was understood as a means of saving money whilst improving community‐based care (National Collaboration and for Care and Support, 2013; Curry and Ham, 2010; Audit Commission, 2011; National Voices, 2013). Reduced use of hospital beds also supported reconfiguration plans. The national policy discourse of integrated care as a means to reduce the need for emergency hospital admissions therefore aligned with the organisational conditions of our case. The Partnership strategy of integrated care sought to reduce demand by providing proactive, preventative care, a more palatable approach for local stakeholders than contentious plans interpreted as restricting provision of healthcare.

The Partnership was the outcome of the interplay between national legislation determining the responsibilities of NHS commissioning organisations (Health & Social Care Act, 2012), the pattern of hospital services which provided the basis for NHS plans, and the engagement of local councils. The resulting configuration offered an example of ‘supraorganisational leadership’, with roles and influences extending beyond individual organisations (Denis et al., 2001). The Partnership concurred on an integrated care strategy that involved collaborative work to coordinate patient care and so reduce hospital admissions and cost, whilst improving care and adhering to local reconfiguration plans. The strategy thus achieved an alignment, or an environmental ‘coupling’ between the local circumstances facing the Partnership and national policy. The NHS and council leaders of the Partnership exercised their agency dynamically as they made decisions about the integrated care strategy; their actions created a strategy that was similar to those developed elsewhere in the UK, shaped by both the local setting and national policy. The strategy itself then shaped how professionals worked in the Partnership; they drew on the strategy to consider solutions to the local challenges.

## Producing a structure of integrated care

The Partnership strategy formed the conditions for the production and practice of a local meso‐structure of integrated care. We identified two interrelated sets of practices which recursively produced this structure: integrated case management (ICM) and financial planning processes. There has been a tendency for UK programmes of integrated care to converge on strategies of case management by multi‐disciplinary teams (Stokes, Checkland and Kristensen, 2016; Erens et al., 2017; Sheaff et al., 2018). Case management, as an intervention of providing better coordinated preventative care, is expected to reduce hospital admissions for patients. To achieve this aim, UK models of integrated care take a targeted approach by risk‐stratifying patients to identify those considered to be at high risk of hospital admission. ICM was described in the Partnership strategy as a tangible example of collaborative working between health and social care that could improve patient care:

*‘…a model of practice which aims to ensure that patients with complex health and social care needs received the right care, in the right place, at the right time.’* [Integrated care strategy document].

ICM was a version of an approach developed by community nurses, social workers and GPs working together in certain geographical ‘clusters’ to coordinate care for people with long‐term conditions. The approach, influenced by the long‐term care model (Department of Health, 2005), had been facilitated by collaborative working relationships. The Partnership decided to standardise the model and roll it out across the whole area. ICM was expected to reduce hospital admissions and so featured as a cost‐saving measure in NHS commissioning plans. We analysed how the model of care and financial planning processes mutually constituted the structure of integrated care.

Standardising and spreading ICM involved promotion and dissemination of the model and inclusion in organisational contracts. Commissioners produced a set of documents to summarise the model of care and held a series of meetings and workshops with health and social care practitioners to make the case for ICM, offering examples of how it had worked well elsewhere. In addition to the work to share knowledge of and to promote the model to the professionals who were expected to deliver it, commissioners and providers worked together to agree the contracts that would formalise how the model of care would be resourced and monitored.

BOX 2 **Composite narrative of integrated case management (ICM)**

*Targeting* *high‐risk patients*

National policy and financial contracts required primary care doctors (GPs) to undertake ‘risk stratification’ to target patients for interventions to reduce hospital admissions.

Commissioners centralised and automated risk stratification by applying an algorithm to all GP electronic patient records collated in a central data warehouse. A risk ‘score’ was allocated to each patient according to factors associated with a high risk of hospital admission (e.g. number of medications, long‐term conditions and smoking status). Patients in the 99^th^ percentile for risk (known locally as the top 1%) were referred to the ICM team.

*Multi‐disciplinary* *teams*

The practitioners personally responsible for providing or organising care for patients on the ICM caseload would meet regularly (namely GP, community matron, social worker, and the liaison officer who organised the meetings and managed the caseloads). Other professionals (e.g. district nurses, mental health workers) would also attend where possible. This team, between them, would have detailed knowledge of their current caseload and would also often have been previously involved in the care of new referrals (who would have been seeing health care professionals regardless of their risk score).

*Care* *planning*

The team would discuss each new referral, share any prior knowledge, and, taking account of the staffing resources available, decide whether or not to accept the patient formally onto their caseload.

If new referrals were already known to the team and considered to be stable, the team might agree there would be no additional benefit in simply adding them to the caseload, as patients would continue to access their GP or other professionals as needed. The patient would not be aware that they have been discussed by the team.

Any patients not known by the team to be ‘stable’ at the point of referral would be accepted and logged as a new case. The liaison officer would organise for the community matron or social worker (depending on what seemed to be the patient’s main presenting need) to visit the patient. At that visit, the professional would assess the patient to identify any actions required to optimise their health and wellbeing and avoid hospital admission (e.g. referrals to other services, medication review or further specialist assessment). Actions would be written up as a care plan.

The patient would stay on the caseload, receiving follow‐up phone calls and visits as needed (and as time and caseloads permitted). When patients were considered to be stable and therefore no longer in need of, or likely to benefit from, ICM, they would be discharged, with the advice that they could call the liaison officer or matron if they ever needed to; patients returning to ICM in this way would be logged as new referrals. Patients would also continue to access any services that they had been referred to, and to see their GP, as needed.

*Monitoring* *performance*

The liaison officer oversaw the ‘RAG (red, amber, green) rating’ of caseloads, with new referrals labelled RED, patients receiving ongoing care flagged as AMBER, and stable patients as GREEN at which point they would be discharged from the active caseload. The officer would also add an electronic copy of the care plan to the patient’s records.

Caseloads, care plans and admissions to hospital were linked to performance monitoring and payment processes, which were written into healthcare provider contracts and into local, regional and national performance ratings. Performance against these targets was monitored by senior officials.

The composite narrative shows how the model of ICM was connected, by contracting arrangements, to financial plans and policy directives. We mapped ICM across four kinds of financial plan during fieldwork: commissioner cost‐saving (Quality Innovation Productivity and Prevention or QIPP) plans, contracts between NHS commissioners and providers, regional financial plans and pooled budgets (known as Better Care Funds) between the NHS and local authorities. We found that ICM was reproduced as a cost‐saving practice through its inclusion in financial plans and the inclusion of ICM in financial plans reproduced the model of care.

ICM was included in a pooled budget known as the Better Care Fund. The Better Care Fund was a directive introduced during fieldwork by two central government departments (the Departments of Health and Communities and Local Government) which required health and social care bodies to create a pooled budget for integrated health and social care ‘schemes’. These schemes (such as ICM) were expected to achieve quantified targets including reductions in admissions to hospitals and fewer delays in discharge from hospital (Departments of Health and Communities and Local Government, 2014).

We observed, and participated in, the work undertaken by council and NHS staff to develop Better Care Funds in workshops, webinars, and meetings which culminated in the completion of two templates: a word document and an excel spreadsheet. The council and NHS officials who completed and approved these documents undertook detailed accounting and narrative work, materially shaped by the pre‐set grids and formulae of the spreadsheet (Dourish, 2017). For example, the Better Care Fund template required each scheme to have a source of funding identified from either health or social care. Although the phrase ‘Better Care Fund’ evoked the idea of new money for integrated care, in reality the funds comprised money already allocated to CCGs and councils and usually committed to paying for contracted or directly provided services. Completing this part of the template involved a process of ‘re‐badging’ existing budget lines. Staff working in the NHS and councils identified existing funding sources and allocated them to the Better Care Fund, producing an integrated budget (at least in principle).

ICM was positioned as the cause of reduced hospital admissions (and other performance targets) through the structure of the ‘benefits plan’. Council and NHS officials selected benefits from a drop‐down menu in the Better Care Fund spreadsheet which listed national targets including reductions of emergency hospital admissions and delayed transfers of care. Officials populated the cell linked to ‘reduced emergency admissions’ with ‘ICM’ to present a causal relationship between ICM as an intervention and the outcome of reduced hospital admissions. A property of the spreadsheet linked the planned reduction in hospital admissions to financial savings; formulae embedded in the spreadsheet converted the number of admissions avoided (calculated in the ICM scheme from the numbers of patients case‐managed) to a percentage change in emergency admissions and thence to expected cost savings. Officials completing the Better Care Fund had to comply with the material requirements of the template to present ICM as a savings scheme and thus reproduced expectations that it would save money.

Pooled budgets were the outcome of the narrative and accounting work of NHS and council commissioners and the structures of the Better Care Fund, manifested in templates which configured ICM in three distinct ways: as the product of integrated funding, as the cause of improved outcomes and a method of saving money. The Better Care Fund presented ICM as an example of integrated health and social care and reproduced expectations of cost savings from integrated care (and specifically from ICM). Cost savings, projected from a containment of acute hospital activity by integrated care, were incorporated into contract negotiations between commissioners and hospital trusts. The saving assumptions that resulted from these negotiations were in turn incorporated into NHS budgets, and through aggregation with neighbouring planning units, into regional financial plans. The balanced budgets that resulted were contingent on integrated care working effectively to prevent people being admitted into hospital. The Partnership strategy therefore shaped the actions of officials as they created the Better Care Fund and reproduced the broader policy of integrating care to improve efficiency across health and social care.

## Professional practices of integrated care

The meso‐structure of integrated care provided the conditions for health and social care practitioners to enact the model of care. We found that as professionals practised ICM, they mediated between external and internal structures of ICM, their professional values, and prior knowledge of local services and patients as they decided how to act.

Discussion about new referrals to ICM at multi‐disciplinary meetings gave us an empirical example of these dynamics; the processes of structuration. As described in Box 2, new referrals were initially identified by a risk stratification process then discussed by the team before being accepted onto the caseload. The external structures of integrated care had made certain resources available including: jobs within the team, regular meetings and risk stratification software. Patients were configured by the automated process of risk stratification as ‘new’ referrals even though they were often already receiving care from members of the team. The team, in discussing patients, were further informed by their conjuncturally specific knowledge (of the ICM caseload and team criteria). The team drew on forms of more general knowledge (of the patient and local services) which enabled them to consider if there would be any benefit to adding the patient to their caseload. Health and social care professionals were strongly oriented to the structures of integrated care, but mediated their actions with recourse to other internal structures. Consideration of new referrals by the ICM team demonstrated an interplay between the agency of the professionals attending, the external structures of ICM and the internal structures of professional knowledge. The outcomes of the meeting included a reproduction and amendment to the external structure of ICM (with a new referral added to the caseload) and events such as new assessments and patient visits. Whilst the ICM team worked to reproduce the model of care, their actions could remain disconnected from patients who were unaware of how they were being referred and ‘managed’, as we examine below.

## Patients’ experiences of integrated care

We set out to understand what the avoidance of hospital admission meant for patients, this entailed a significant switch of focus from organisational and professional concerns. From ethnographic fieldwork, we analysed to what extent the structures of integrated care shaped how patients maintained their *position–practice;* how patients actively created and were able to inhabit their homes through and in social and organisational networks (Greenhalgh and Stones, 2010). We concluded that whilst health and social care services were necessary components of these networks, the specific structures of integrated care were insufficient to compensate for changes in patients’ ability to draw on the resources that sustained them at home.

We provide a vignette of one patient, Alf, to show how he lived independently at home by drawing on a range of social networks and material resources. His ability to do so was affected by health conditions which impaired his physical abilities, causing him to adapt and modify his home accordingly. When Alf became incapacitated following a fall, he was no longer able to sustain his position–practice. Whilst Alf’s situation was unique, his experiences demonstrate common processes observed across our fieldwork. The events described in this vignette were directly observed and participated in during fieldwork.

*“Alf had lived alone since his wife had died. He had significant health issues which caused him to experience tiredness, breathlessness, pain and stiffness in his hands and back and ongoing dizziness. Alf was unsteady on his feet and didn’t leave his house alone. He lived mainly downstairs, having had his bed moved into his living room after a fall on his stairs. He spent his time watching TV and pottering round his kitchen, going out into his back yard to feed the pigeons. He was fiercely independent, rejecting the idea of a ‘stranger’ to come in and help him with cooking, dressing or cleaning. His eldest daughter (who lived a short bus ride away) would check up on him most mornings – ostensibly for a cup of coffee – and fetched his supermarket shopping. His grandsons did odd jobs around the house. Alf supplemented his supermarket shop with ready‐made delivered meals. He was on the ICM caseload due to his high risk of hospital admission, his community matron contacted him regularly, and he had annual outpatient appointments to monitor his cardiac and renal problems*.

*Alf fell early one morning in the kitchen at the back of his house, hitting his head which bled profusely. Dazed, he couldn’t stand. After lying on the floor for a while, he reached the phone fixed to the kitchen wall. Without his glasses on, he was only able to dial 999. An ambulance was dispatched. On arrival, the ambulance crew found the front door locked, Alf still lying in the kitchen. The crew mobilised the police to gain access, but then Alf’s granddaughter arrived with a spare key – she’d been sent by Alf’s daughter who was unwell. The ambulance crew followed their protocol to take Alf to hospital where he was assessed and admitted as an emergency. Alternative community‐based treatments were not appropriate given the nature of his head injury*.”

Alf’s embodied experiences were typical of older patients we observed who narrated their conditions as linked to the processes of ageing, with common experiences of pain, breathlessness, fatigue and loss of balance. The result for most of these patients was significantly reduced mobility, often passing their time within a few metres of space within their homes. Patients commonly adapted their material environments to compensate; upstairs became out of bounds and household objects were pressed into service alongside occupational therapy‐supplied aids and adaptations.

The resources offered by the structures of integrated care contributed to patients’ ability to live at home, for example regular monitoring of symptoms, medication reviews, arranging domiciliary care. A case manager would offer regular support; long‐standing patients (like Alf) would get a monthly phone call. These contributions were often of limited relevance and visibility to patients, most of whom needed daily support to manage their long‐term conditions at home. Further, the coordinating activities of the ICM team were not obvious to patients who continued to access a complex array of specialist services (such as community heart failure and respiratory teams, the ‘warfarin nurse’, and hospital outpatients) and at times reported a distinct lack of coordination, for example on discharge from hospital. ICM team members, in substituting for or referring to other services, offered only marginal gains for patients over other ways of accessing care (for example a community matron could adjust a prescription or arrange for district nurses to dress a wound rather than the patient needing to visit their GP).

Patients secured their daily support through a process best characterised as ‘bricolage’ or a ‘making do with tools that are available to address an immediate, local and contingent problem or need…..crafting solutions with whatever is at hand’ (Greenhalgh et al., 2013 p93). Bricolage has been used to characterise both how people use (or abandon) assisted living technologies and how people access different resources in addressing their health concerns (Phillimore et al., 2019). Alf, in the vignette above, was able to live at home by drawing on a complex network of support. He created his own order and logic out of the resources available, for example choosing not to accept paid domiciliary care but accepting support from his daughter.

Living at home with multiple long‐term conditions was, for these patients, the outcome of effortful accomplishment of daily life contingent on their ability to draw on the material resources, practical help and social support to hand. Alf’s fall exposed the fragility and interdependence of his social, material and physical position–practice at home. External structures of family and integrated care were neither available to Alf as he fell, nor could they compensate for his injury and reduced material circumstances. Instead, the external structures and the physical intervention of the emergency services were required.

In sum, our findings show how policy directives, organisational responses and practitioners’ actions were connected across a field of practices to dynamically produce and reproduce structures of integrated care, and how these structures were of limited relevance and availability to patients’ daily lives. Policy directives were incorporated into our case through strategic plans which shaped resource allocations, and expectations about how services should be organised. The leaders of organisations interpreted these directives, which were moderated by local decisions and priorities, to change how services were organised. A structure of integrated care emerged as and from a set of practices to produce a model of care which would deliver cost savings. Integrated care contributed towards the complex practices that resulted in the outcome of patients living at home, but provided insufficient resources to compensate for sudden changes in patients’ abilities to mobilise the networks that sustained their position–practice.

## DISCUSSION

Our analysis of the empirical processes of structuration across the field of practices of integrated care provides new insights into the nature of integrated care, why integrated care interventions might not achieve their desired outcomes, and the role of embodied and materially shaped practices in relation to outcomes of processes of structuration. Our study was limited, empirically, to a small number of patients; however, what became visible from the patients’ vantage point was the complexity of the social practices and embodied, material contexts that contributed to their ability to manage at home with multiple long‐term conditions.

SST provided a theoretical explanation for how the structures of integrated care were recursively produced by and created the conditions for processes of structuration. Integrated care existed autonomously to individual actors in our case as a set of external structures articulated and reproduced in a model of care, contracts, performance targets and templates all of which were understood as logical responses to, and reinforcing the idea of, integrated care as a cost‐saving approach to improve care and reduce hospital admissions. We offer an analogy of integrated care as being a structure which is like a ‘lens’ creating a normative view of health and social care services. In our case of integrated care, this lens was an internal structure shaping how professionals acted, orienting them towards the contextually important outcomes of avoiding hospital admissions. The lens of integrated care was used across the field of practices, in strategy development, financial planning and ICM meetings, but was not available to patients. Integrated care was also an external structure, a resource in the shape of strategies, plans and a model of care that brought into focus certain interactions between patients and services (such as care planning processes oriented to coordinating care and preventing hospital admissions). The lens of integrated care made avoidance of hospital admission (for patients at risk) seems possible from an organisational and professional perspective, but it did not bring into view the wider network of resources patients drew on to maintain their position–practices.

Integrated care acted primarily as a lens (a planning, preparatory and epistemological resource) for professionals organising care and not as a material resource for patients needing an emergency response to a sudden change in circumstances. The desired outcome of avoiding hospital admission was contingent on material features of patients’ homes, their embodied health and their structures of support. Materiality and embodied agency were necessary resources to the outcomes of patients’ position–practices distinct from internal and external social structures—patients were able to manage their long‐term conditions at home because of their ability to mobilise networks of support. We offer this insight to extend our understanding of how the processes of structuration, in producing new structures, might (or might not) produce different outcomes. Material and embodied resources need to be available at the point of intersection between structure and agency for material, embodied outcomes to be affected by the dynamics of structuration.

## CONCLUSION

Our concern to develop a theoretically informed explanation of why integrated care programmes often fail to achieve their desired aims led us to use SST as a synthesising theoretical framework to analyse an empirical case study. We identified processes of structuration that accounted for connections between the different networks of human action and structures that we observed across our field of practices.

Integrated care was recursively reproduced in our case as internal and external structures (observed in the enactment of ICM and the production of the BCF), which we liken to a lens through which interactions between patients and services were viewed. This lens offered the possibility of outcomes that were contextually significant: avoiding hospital admissions. However, the structures of integrated care were only a part of the complex network of resources patients drew on in managing their long‐term conditions at home and were insufficiently available or effective in compensating for bodily and material changes that affected patients’ health. For integrated care to work (for structures to affect material, embodied outcomes), patients need to be able to draw on relevant and timely resources.

## AUTHOR CONTRIBUTIONS

**Gemma Hughes:** Conceptualization‐Lead, Data curation‐Lead, Formal analysis‐Lead, Investigation‐Lead, Methodology‐Equal, Project administration‐Lead, Writing‐original draft‐Lead; **Sara E. Shaw:** Conceptualization‐Supporting, Formal analysis‐Supporting, Funding acquisition‐Supporting, Investigation‐Supporting, Methodology‐Equal, Supervision‐Equal, Writing‐review & editing‐Equal; **Trish Greenhalgh:** Conceptualization‐Supporting, Formal analysis‐Supporting, Funding acquisition‐Lead, Investigation‐Supporting, Methodology‐Equal, Supervision‐Equal, Writing‐review & editing‐Equal.

## Data Availability

The data that support the findings of this study are available from the corresponding author upon reasonable request.
